# *endo*-11-(Dibenzyl­amino)­tetra­cyclo­[5.4.0.0^3,10^.0^5,9^]undecane-8-one

**DOI:** 10.1107/S160053681100479X

**Published:** 2011-02-12

**Authors:** Rajshekhar Karpoormath, Patrick Govender, Thavendran Govender, Hendrik G. Kruger, Glenn E. M. Maguire

**Affiliations:** a School of Chemistry, University of KwaZulu-Natal, Durban 4000, South Africa; b Department of Biochemistry, University of KwaZulu-Natal, Durban 4000, South Africa; c School of Pharmacy and Pharmacology, University of KwaZulu-Natal, Durban 4000, South Africa

## Abstract

The structure of the title compound, C_25_H_27_NO, is a mono-ketone penta­cyclo­undecane (PCU) mol­ecule bearing a tertiary amine group. One of the methyl­ene groups in the PCU is disordered over two orientations with site-occupancy factors of 0.621 (7) and 0.379 (7).

## Related literature

For mono-ketone PCU derivatives, see: Kruger *et al.* (2006) ▶. For examples of the crystal structures of mono-ketone PCU mol­ecules bearing heteroatoms, see: Watson *et al.* (2000) ▶; Karpoormath *et al.* (2010 ▶).
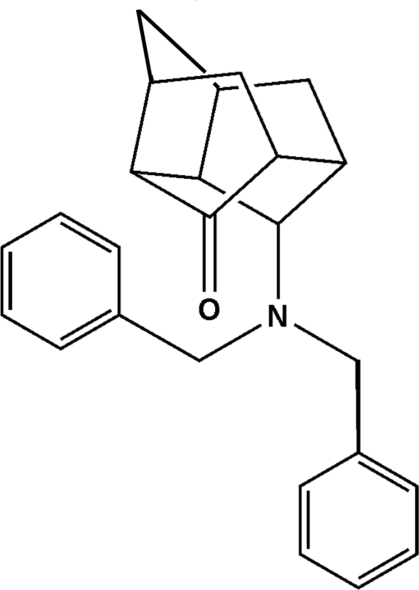


## Experimental

### 

#### Crystal data

C_25_H_21_NO
*M* *_r_* = 351.43
Monoclinic, 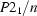

*a* = 6.6117 (3) Å
*b* = 16.4344 (7) Å
*c* = 17.2331 (8) Å
β = 97.100 (2)°
*V* = 1858.18 (14) Å^3^
*Z* = 4
Cu *K*α radiation
μ = 0.59 mm^−1^
*T* = 173 K
0.43 × 0.33 × 0.25 mm

#### Data collection

Bruker Kappa DUO APEXII diffractometer
Absorption correction: multi-scan (*SADABS*; Bruker, 2006 ▶) *T*_min_ = 0.786, *T*_max_ = 0.867
24662 measured reflections
3303 independent reflections
3240 reflections with *I* > 2σ(*I*)
*R*_int_ = 0.018

#### Refinement

*R*[*F* ^2^ > 2σ(*F* ^2^)] = 0.066
*wR*(*F* ^2^) = 0.173
*S* = 1.06
3303 reflections
255 parameters
24 restraints
H-atom parameters constrained
Δρ_max_ = 0.48 e Å^−3^
Δρ_min_ = −0.46 e Å^−3^

### 

Data collection: *APEX2* (Bruker, 2006 ▶); cell refinement: *SAINT* (Bruker, 2006 ▶); data reduction: *SAINT*; program(s) used to solve structure: *SHELXS97* (Sheldrick, 2008 ▶); program(s) used to refine structure: *SHELXL97* (Sheldrick, 2008 ▶); molecular graphics: *X-SEED* (Barbour, 2001 ▶); software used to prepare material for publication: *SHELXL97*.

## Supplementary Material

Crystal structure: contains datablocks I, global. DOI: 10.1107/S160053681100479X/lx2177sup1.cif

Structure factors: contains datablocks I. DOI: 10.1107/S160053681100479X/lx2177Isup2.hkl

Additional supplementary materials:  crystallographic information; 3D view; checkCIF report

## Figures and Tables

**Fig. 1.**
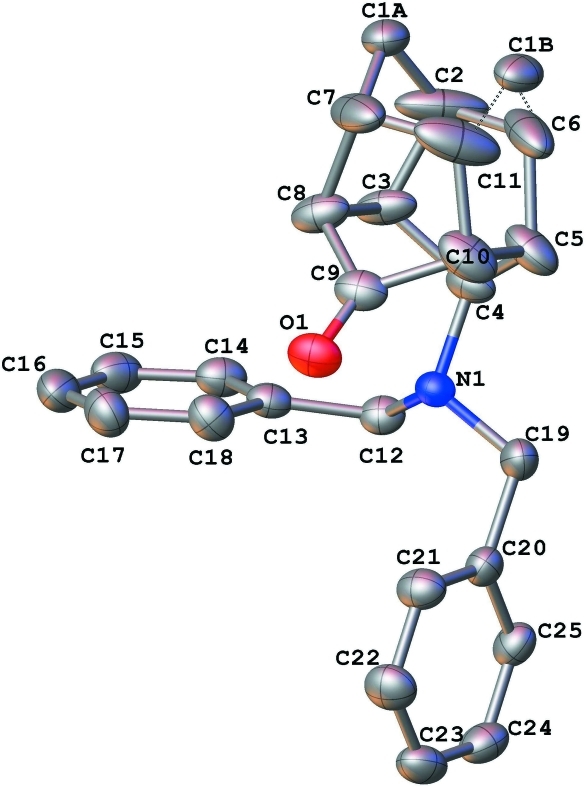
The molecular structure of the title compound with atomic numbering scheme. All hydrogen atoms are omitted for clarity. Displacement ellipsoids are drawn at the 25% probability level. The C1 methylene group was found to be disordered over two positions and modelled with site–occupancy factors, from refinement of 0.621 (7) (C1A) and 0.379 (7) (C1B).

## supplementary crystallographic information

### Comment

We have reported the structures of a number of PCU derivatives including a mono-ketone ethylene acetal (Kruger *et al.*, 2006). We more recently reported the structure of a mono–ketone pentacycloundecane (PCU) (Karpoormath *et al.* 2010), that demonstrated intramolecular hydrogen bonding, a quite uncommon feature hitherto in mono–ketone PCU structures (Watson *et al.*, 2000). In that example the racemate occupied alternative sites in the unit cell.

Herein, we report the crystal structure of the title compound (Fig. 1). The C1 methylene group in PCU is disordered over two positions with site–occupancy factors of 0.621 (7) (for atom labelled A) and 0.379 (7) (for atom labelled B) in Fig. 1.

### Experimental

A solution of PCU cage *N*-dibenzyl mono ethylene ketal (0.5 g, 1.25 mmol) in 10 ml of THF was stirred at room temperature for 5 minutes. To this mixture was added 10 ml of 10% HCL solution and stirred overnight at room temperature. THF was removed from the crude product under vacuum using a teflon pump at 80 °C to obtain an aqueous solution with white precipitate. The precipitate was collected by vacuum filtration and washed with water (50 ml) to give a white solid. The yield was 97%. Crystallization of the title compound was carried out by dissolving the compound in ethyl acetate and hexane (1:4) with storage at 20 °C. Melting point: 438–439 K. IR (neat) Vmax cm^-1^: 3376.61, 2978.73, 2961.26, 2794.11, 1721.74, 1602.42, 1494.57, 1342.20, 1131.34, 752.25, 731.39, 696.73, cm^-1^. ^1^H NMR (CDCl_3_, 400 MHz) δ p.p.m.: 1.56 (1.0H, d, J=11.13 Hz), 1.89 (1.0H, d, J=11.13 Hz), 2.52 (1.0H, d, J=4.56 Hz), 2.61 (1.0H, d, J=6.96 Hz), 2.71 (2.0H, d, J=5.96 Hz), 2.90 (1.0H, d, J=5.60 Hz), 2.93 (1.0H, d, J=4.68 Hz), 3.50 (1.0H, d, J=2.28 Hz), 3.51 (2.0H, t, J=12.27 Hz), 3.90 (1.0H, t, J=5.02 Hz), 4.39 (1.0H, d, J=14.65 Hz), 4.52 (2.0H, dd, J=9.87, 14.55 Hz), 4.81 (1.0H, d, J=14.61 Hz), 6.90 (2.0H, d, J=7.24 Hz), 6.99 (2.0H, d, J=7.20 Hz), 7.23 - 7.38 (6.0H, m, J=7.20 Hz). ^13^C NMR (CDCl_3_, 101 MHz) δ p.p.m.: 40.75 (d, *J*=15.71 Hz), 41.28 (d, *J*=14.29 Hz), 43.15 (*s*), 44.18 (*s*), 46.12 (*s*), 50.09 (*s*), 52.72 (*s*), 59.20 (d, *J*=73.03 Hz), 70.28 (*s*), 123.86 (*s*), 129.41 (d, J=10.10 Hz), 129.92 (*s*), 130.45 (d, *J*=24.83 Hz), 131.13 (*s*).

HR ESI m/*z*: calcd for C_25_H_25_NO [*M*+H]^+^:356.2009 found 356.2014.

### Refinement

All hydrogen atoms were positioned geometrically with C—H = 0.95–1.00 Å and refined as riding on their parent atoms, with *U*_iso_ (H) = 1.2 *U*_eq_ (C). The C1 methylene group was found to be disordered over two positions and modelled with site–occupancy factors, from refinement of 0.621 (7) (C1A) and 0.379 (7) (C1B), respectively. The distance of C2—C1A, C6—C1B and C7—C1A and C11—C1B  sets were restrained to 0.001 Å using command SADI and DELU. The displacement ellipsoids of C1A and C1B were restrained using commend ISOR (0.01).

### Crystal data

**Table:**

C_25_H_21_NO	*F*(000) = 744
*M**_r_* = 351.43	*D*_x_ = 1.256 Mg m^−^^3^
Monoclinic, *P*2_1_/*n*	Cu *K*α radiation, λ = 1.54184 Å
Hall symbol: -P 2yn	Cell parameters from 3299 reflections
*a* = 6.6117 (3) Å	θ = 5.2–69.2°
*b* = 16.4344 (7) Å	µ = 0.59 mm^−^^1^
*c* = 17.2331 (8) Å	*T* = 173 K
β = 97.100 (2)°	Needle, colourless
*V* = 1858.18 (14) Å^3^	0.43 × 0.33 × 0.25 mm
*Z* = 4	

### Data collection

**Table:**

Bruker Kappa DUO APEXII diffractometer	3303 independent reflections
Radiation source: fine-focus sealed tube	3240 reflections with *I* > 2σ(*I*)
graphite	*R*_int_ = 0.018
1.2° φ scans and ω	θ_max_ = 69.2°, θ_min_ = 3.7°
Absorption correction: multi-scan (*SADABS*; Bruker, 2006)	*h* = −7→7
*T*_min_ = 0.786, *T*_max_ = 0.867	*k* = −19→19
24662 measured reflections	*l* = −20→20

### Refinement

**Table:**

Refinement on *F*^2^	Secondary atom site location: difference Fourier map
Least-squares matrix: full	Hydrogen site location: difference Fourier map
*R*[*F*^2^ > 2σ(*F*^2^)] = 0.066	H-atom parameters constrained
*wR*(*F*^2^) = 0.173	*w* = 1/[σ^2^(*F*_o_^2^) + (0.080*P*)^2^ + 1.983*P*] where *P* = (*F*_o_^2^ + 2*F*_c_^2^)/3
*S* = 1.06	(Δ/σ)_max_ < 0.001
3303 reflections	Δρ_max_ = 0.48 e Å^−^^3^
255 parameters	Δρ_min_ = −0.46 e Å^−^^3^
24 restraints	Extinction correction: *SHELXL97* (Sheldrick, 2008), Fc^*^=kFc[1+0.001xFc^2^λ^3^/sin(2θ)]^-1/4^
Primary atom site location: structure-invariant direct methods	Extinction coefficient: 0.0020 (4)

### Special details

**Table:**

Experimental. Half sphere of data collected using *APEX2* (Bruker, 2006). Crystal to detector distance = 45 mm; combination of φ and ω scans of 1.2°, 50 s per °, 2 iterations.
Geometry. All esds (except the esd in the dihedral angle between two l.s. planes) are estimated using the full covariance matrix. The cell esds are taken into account individually in the estimation of esds in distances, angles and torsion angles; correlations between esds in cell parameters are only used when they are defined by crystal symmetry. An approximate (isotropic) treatment of cell esds is used for estimating esds involving l.s. planes.
Refinement. Refinement of F^2^ against ALL reflections. The weighted R-factor wR and goodness of fit S are based on F^2^, conventional R-factors R are based on F, with F set to zero for negative F^2^. The threshold expression of F^2^ > 2sigma(F^2^) is used only for calculating R-factors(gt) etc. and is not relevant to the choice of reflections for refinement. R-factors based on F^2^ are statistically about twice as large as those based on F, and R- factors based on ALL data will be even larger.

### Fractional atomic coordinates and isotropic or equivalent isotropic displacement parameters (Å^2^)

**Table:**

	*x*	*y*	*z*	*U*_iso_*/*U*_eq_	Occ. (<1)
O1	0.1742 (2)	0.13889 (10)	0.19402 (10)	0.0394 (4)	
N1	−0.2672 (3)	0.05165 (10)	0.14408 (10)	0.0271 (4)	
C2	−0.4175 (5)	0.27147 (16)	0.1215 (3)	0.0763 (12)	
C3	−0.3558 (3)	0.19546 (14)	0.17218 (17)	0.0423 (6)	
H3	−0.4438	0.1857	0.2145	0.051*	
C4	−0.3624 (3)	0.12730 (13)	0.11264 (14)	0.0347 (5)	
H4	−0.5077	0.1160	0.0920	0.042*	
C5	−0.2577 (4)	0.16781 (17)	0.04842 (17)	0.0493 (7)	
H5	−0.2759	0.1387	−0.0029	0.059*	
C6	−0.3393 (6)	0.25559 (17)	0.04539 (18)	0.0652 (10)	
C7	−0.1306 (5)	0.30438 (16)	0.16140 (19)	0.0612 (9)	
C8	−0.1293 (3)	0.22060 (14)	0.20361 (17)	0.0422 (6)	
H8	−0.0991	0.2233	0.2619	0.051*	
C9	0.0227 (3)	0.17342 (14)	0.16393 (15)	0.0361 (5)	
C10	−0.0309 (4)	0.19225 (18)	0.07827 (17)	0.0497 (7)	
H10	0.0730	0.1760	0.0437	0.060*	
C11	−0.0792 (5)	0.2836 (2)	0.0801 (2)	0.0775 (12)	
C12	−0.3867 (3)	0.01601 (13)	0.20230 (13)	0.0311 (5)	
H12A	−0.3795	−0.0440	0.1982	0.037*	
H12B	−0.5311	0.0318	0.1884	0.037*	
C13	−0.3230 (3)	0.03943 (13)	0.28639 (13)	0.0307 (5)	
C14	−0.4688 (4)	0.04214 (14)	0.33779 (14)	0.0372 (5)	
H14	−0.6085	0.0347	0.3185	0.045*	
C15	−0.4127 (4)	0.05563 (15)	0.41683 (15)	0.0436 (6)	
H15	−0.5139	0.0568	0.4514	0.052*	
C16	−0.2108 (4)	0.06739 (16)	0.44562 (15)	0.0467 (6)	
H16	−0.1722	0.0761	0.4999	0.056*	
C17	−0.0654 (4)	0.06631 (17)	0.39452 (15)	0.0452 (6)	
H17	0.0736	0.0755	0.4138	0.054*	
C18	−0.1200 (4)	0.05196 (15)	0.31545 (14)	0.0384 (6)	
H18	−0.0184	0.0507	0.2810	0.046*	
C19	−0.2474 (4)	−0.00746 (14)	0.08166 (13)	0.0349 (5)	
H19A	−0.1791	0.0190	0.0403	0.042*	
H19B	−0.3851	−0.0244	0.0580	0.042*	
C20	−0.1277 (3)	−0.08185 (13)	0.11058 (12)	0.0312 (5)	
C21	0.0737 (3)	−0.07388 (14)	0.14401 (14)	0.0381 (5)	
H21	0.1342	−0.0214	0.1496	0.046*	
C22	0.1864 (4)	−0.14184 (16)	0.16920 (16)	0.0434 (6)	
H22	0.3238	−0.1357	0.1921	0.052*	
C23	0.1013 (4)	−0.21862 (15)	0.16137 (15)	0.0416 (6)	
H23	0.1796	−0.2652	0.1785	0.050*	
C24	−0.0992 (4)	−0.22698 (15)	0.12834 (15)	0.0413 (6)	
H24	−0.1593	−0.2795	0.1228	0.050*	
C25	−0.2121 (4)	−0.15900 (14)	0.10333 (14)	0.0363 (5)	
H25	−0.3498	−0.1653	0.0808	0.044*	
C1A	−0.3205 (6)	0.3417 (2)	0.1498 (2)	0.0414 (11)	0.621 (7)
H1A	−0.3673	0.3612	0.1989	0.050*	0.621 (7)
H1B	−0.3281	0.3860	0.1106	0.050*	0.621 (7)
C1B	−0.2299 (8)	0.3246 (3)	0.0321 (4)	0.0458 (19)	0.379 (7)
H1D	−0.2791	0.3753	0.0546	0.055*	0.379 (7)
H1C	−0.2020	0.3321	−0.0225	0.055*	0.379 (7)

### Atomic displacement parameters (Å^2^)

**Table:**

	*U*^11^	*U*^22^	*U*^33^	*U*^12^	*U*^13^	*U*^23^
O1	0.0224 (8)	0.0389 (9)	0.0564 (10)	0.0003 (6)	0.0031 (7)	0.0051 (7)
N1	0.0261 (9)	0.0239 (9)	0.0310 (9)	0.0000 (7)	0.0019 (7)	0.0019 (7)
C2	0.0606 (19)	0.0306 (14)	0.148 (3)	0.0175 (13)	0.055 (2)	0.0285 (18)
C3	0.0238 (11)	0.0256 (12)	0.0770 (18)	0.0021 (9)	0.0049 (11)	−0.0047 (11)
C4	0.0248 (10)	0.0278 (11)	0.0495 (13)	0.0006 (8)	−0.0037 (9)	0.0082 (10)
C5	0.0439 (14)	0.0489 (16)	0.0522 (15)	−0.0049 (12)	−0.0061 (12)	0.0247 (12)
C6	0.092 (2)	0.0341 (14)	0.0589 (17)	−0.0159 (15)	−0.0342 (16)	0.0170 (13)
C7	0.0715 (19)	0.0268 (13)	0.075 (2)	0.0005 (12)	−0.0338 (16)	−0.0049 (13)
C8	0.0278 (12)	0.0284 (12)	0.0699 (17)	−0.0016 (9)	0.0041 (11)	−0.0090 (11)
C9	0.0243 (11)	0.0292 (11)	0.0549 (14)	−0.0052 (9)	0.0055 (10)	0.0056 (10)
C10	0.0382 (13)	0.0525 (16)	0.0595 (16)	−0.0045 (11)	0.0098 (12)	0.0249 (13)
C11	0.066 (2)	0.059 (2)	0.117 (3)	0.0220 (16)	0.050 (2)	0.052 (2)
C12	0.0262 (10)	0.0269 (11)	0.0404 (12)	−0.0041 (8)	0.0055 (9)	0.0009 (9)
C13	0.0311 (11)	0.0240 (10)	0.0378 (12)	0.0013 (8)	0.0069 (9)	0.0060 (9)
C14	0.0361 (12)	0.0309 (12)	0.0464 (13)	0.0036 (9)	0.0124 (10)	0.0049 (10)
C15	0.0570 (16)	0.0351 (13)	0.0423 (13)	0.0077 (11)	0.0206 (12)	0.0067 (10)
C16	0.0684 (18)	0.0373 (13)	0.0338 (12)	0.0072 (12)	0.0040 (12)	0.0047 (10)
C17	0.0445 (14)	0.0483 (15)	0.0405 (13)	0.0008 (11)	−0.0041 (11)	0.0043 (11)
C18	0.0327 (12)	0.0435 (14)	0.0391 (13)	0.0013 (10)	0.0053 (10)	0.0051 (10)
C19	0.0376 (12)	0.0351 (12)	0.0301 (11)	0.0035 (9)	−0.0027 (9)	−0.0024 (9)
C20	0.0345 (11)	0.0303 (11)	0.0287 (10)	0.0012 (9)	0.0032 (9)	−0.0043 (9)
C21	0.0333 (12)	0.0302 (12)	0.0498 (14)	−0.0017 (9)	0.0011 (10)	−0.0011 (10)
C22	0.0316 (12)	0.0429 (14)	0.0542 (15)	0.0035 (10)	−0.0005 (11)	0.0025 (11)
C23	0.0460 (14)	0.0342 (13)	0.0459 (14)	0.0110 (10)	0.0104 (11)	0.0051 (10)
C24	0.0497 (14)	0.0287 (12)	0.0467 (14)	−0.0026 (10)	0.0111 (11)	−0.0053 (10)
C25	0.0351 (12)	0.0348 (12)	0.0384 (12)	−0.0025 (9)	0.0020 (9)	−0.0085 (10)
C1A	0.052 (2)	0.0290 (19)	0.043 (2)	0.0039 (16)	0.0042 (17)	0.0013 (15)
C1B	0.062 (4)	0.031 (3)	0.046 (3)	0.002 (3)	0.013 (3)	0.005 (3)

### Geometric parameters (Å, °)

**Table:**

O1—C9	1.211 (3)	C13—C14	1.388 (3)
N1—C4	1.467 (3)	C14—C15	1.385 (4)
N1—C19	1.467 (3)	C14—H14	0.9500
N1—C12	1.473 (3)	C15—C16	1.379 (4)
C2—C1A	1.380 (4)	C15—H15	0.9500
C2—C6	1.491 (5)	C16—C17	1.382 (4)
C2—C3	1.550 (4)	C16—H16	0.9500
C3—C4	1.516 (3)	C17—C18	1.386 (4)
C3—C8	1.583 (3)	C17—H17	0.9500
C3—H3	1.0000	C18—H18	0.9500
C4—C5	1.529 (3)	C19—C20	1.507 (3)
C4—H4	1.0000	C19—H19A	0.9900
C5—C6	1.539 (4)	C19—H19B	0.9900
C5—C10	1.576 (4)	C20—C25	1.385 (3)
C5—H5	1.0000	C20—C21	1.390 (3)
C6—C1B	1.379 (4)	C21—C22	1.383 (3)
C7—C1A	1.389 (4)	C21—H21	0.9500
C7—C11	1.522 (5)	C22—C23	1.381 (4)
C7—C8	1.557 (4)	C22—H22	0.9500
C8—C9	1.500 (3)	C23—C24	1.383 (4)
C8—H8	1.0000	C23—H23	0.9500
C9—C10	1.507 (4)	C24—C25	1.383 (3)
C10—C11	1.536 (4)	C24—H24	0.9500
C10—H10	1.0000	C25—H25	0.9500
C11—C1B	1.387 (4)	C1A—H1A	0.9900
C12—C13	1.508 (3)	C1A—H1B	0.9900
C12—H12A	0.9900	C1B—H1D	0.9900
C12—H12B	0.9900	C1B—H1C	0.9900
C13—C18	1.388 (3)		
			
C4—N1—C19	111.32 (17)	C18—C13—C14	118.8 (2)
C4—N1—C12	110.30 (16)	C18—C13—C12	121.8 (2)
C19—N1—C12	109.88 (17)	C14—C13—C12	119.2 (2)
C1A—C2—C6	105.1 (3)	C15—C14—C13	120.7 (2)
C1A—C2—C3	113.4 (3)	C15—C14—H14	119.7
C6—C2—C3	105.0 (2)	C13—C14—H14	119.7
C4—C3—C2	103.3 (2)	C16—C15—C14	120.4 (2)
C4—C3—C8	111.81 (19)	C16—C15—H15	119.8
C2—C3—C8	98.9 (2)	C14—C15—H15	119.8
C4—C3—H3	113.8	C15—C16—C17	119.2 (2)
C2—C3—H3	113.8	C15—C16—H16	120.4
C8—C3—H3	113.8	C17—C16—H16	120.4
N1—C4—C3	113.65 (19)	C16—C17—C18	120.8 (2)
N1—C4—C5	115.1 (2)	C16—C17—H17	119.6
C3—C4—C5	101.0 (2)	C18—C17—H17	119.6
N1—C4—H4	108.9	C17—C18—C13	120.2 (2)
C3—C4—H4	108.9	C17—C18—H18	119.9
C5—C4—H4	108.9	C13—C18—H18	119.9
C4—C5—C6	104.2 (2)	N1—C19—C20	112.67 (17)
C4—C5—C10	111.9 (2)	N1—C19—H19A	109.1
C6—C5—C10	95.0 (2)	C20—C19—H19A	109.1
C4—C5—H5	114.6	N1—C19—H19B	109.1
C6—C5—H5	114.6	C20—C19—H19B	109.1
C10—C5—H5	114.6	H19A—C19—H19B	107.8
C1B—C6—C2	104.3 (4)	C25—C20—C21	118.6 (2)
C1B—C6—C5	126.0 (4)	C25—C20—C19	121.6 (2)
C2—C6—C5	107.0 (2)	C21—C20—C19	119.8 (2)
C1A—C7—C11	105.5 (3)	C22—C21—C20	120.4 (2)
C1A—C7—C8	114.2 (3)	C22—C21—H21	119.8
C11—C7—C8	104.1 (2)	C20—C21—H21	119.8
C9—C8—C7	102.1 (2)	C23—C22—C21	120.6 (2)
C9—C8—C3	111.6 (2)	C23—C22—H22	119.7
C7—C8—C3	96.9 (2)	C21—C22—H22	119.7
C9—C8—H8	114.7	C22—C23—C24	119.3 (2)
C7—C8—H8	114.7	C22—C23—H23	120.4
C3—C8—H8	114.7	C24—C23—H23	120.4
O1—C9—C8	127.8 (2)	C25—C24—C23	120.1 (2)
O1—C9—C10	126.7 (2)	C25—C24—H24	120.0
C8—C9—C10	104.5 (2)	C23—C24—H24	120.0
C9—C10—C11	101.8 (3)	C24—C25—C20	121.0 (2)
C9—C10—C5	111.5 (2)	C24—C25—H25	119.5
C11—C10—C5	93.7 (2)	C20—C25—H25	119.5
C9—C10—H10	115.7	C2—C1A—C7	93.1 (3)
C11—C10—H10	115.7	C2—C1A—H1A	113.1
C5—C10—H10	115.7	C7—C1A—H1A	113.1
C1B—C11—C7	102.3 (4)	C2—C1A—H1B	113.1
C1B—C11—C10	126.7 (4)	C7—C1A—H1B	113.1
C7—C11—C10	108.0 (2)	H1A—C1A—H1B	110.5
N1—C12—C13	116.31 (17)	C6—C1B—C11	81.7 (3)
N1—C12—H12A	108.2	C6—C1B—H1D	115.0
C13—C12—H12A	108.2	C11—C1B—H1D	115.0
N1—C12—H12B	108.2	C6—C1B—H1C	115.0
C13—C12—H12B	108.2	C11—C1B—H1C	115.0
H12A—C12—H12B	107.4	H1D—C1B—H1C	112.1
			
C1A—C2—C3—C4	−145.8 (3)	C4—C5—C10—C11	−107.2 (3)
C6—C2—C3—C4	−31.6 (3)	C6—C5—C10—C11	0.3 (3)
C1A—C2—C3—C8	−30.7 (3)	C1A—C7—C11—C1B	10.1 (3)
C6—C2—C3—C8	83.4 (3)	C8—C7—C11—C1B	130.7 (3)
C19—N1—C4—C3	−170.57 (19)	C1A—C7—C11—C10	−125.5 (3)
C12—N1—C4—C3	67.2 (2)	C8—C7—C11—C10	−4.9 (3)
C19—N1—C4—C5	−54.8 (3)	C9—C10—C11—C1B	−142.8 (4)
C12—N1—C4—C5	−177.03 (19)	C5—C10—C11—C1B	−29.9 (5)
C2—C3—C4—N1	167.47 (19)	C9—C10—C11—C7	−21.3 (3)
C8—C3—C4—N1	62.0 (3)	C5—C10—C11—C7	91.6 (3)
C2—C3—C4—C5	43.6 (2)	C4—N1—C12—C13	−92.8 (2)
C8—C3—C4—C5	−61.8 (2)	C19—N1—C12—C13	144.12 (18)
N1—C4—C5—C6	−162.24 (19)	N1—C12—C13—C18	−35.2 (3)
C3—C4—C5—C6	−39.4 (2)	N1—C12—C13—C14	150.2 (2)
N1—C4—C5—C10	−60.8 (3)	C18—C13—C14—C15	−1.1 (3)
C3—C4—C5—C10	62.1 (3)	C12—C13—C14—C15	173.7 (2)
C1A—C2—C6—C1B	−8.8 (3)	C13—C14—C15—C16	0.6 (4)
C3—C2—C6—C1B	−128.7 (3)	C14—C15—C16—C17	0.6 (4)
C1A—C2—C6—C5	126.6 (3)	C15—C16—C17—C18	−1.3 (4)
C3—C2—C6—C5	6.7 (3)	C16—C17—C18—C13	0.8 (4)
C4—C5—C6—C1B	143.1 (4)	C14—C13—C18—C17	0.4 (4)
C10—C5—C6—C1B	29.1 (5)	C12—C13—C18—C17	−174.3 (2)
C4—C5—C6—C2	20.3 (3)	C4—N1—C19—C20	173.10 (18)
C10—C5—C6—C2	−93.7 (3)	C12—N1—C19—C20	−64.4 (2)
C1A—C7—C8—C9	144.0 (3)	N1—C19—C20—C25	120.5 (2)
C11—C7—C8—C9	29.5 (3)	N1—C19—C20—C21	−60.8 (3)
C1A—C7—C8—C3	30.1 (3)	C25—C20—C21—C22	0.1 (4)
C11—C7—C8—C3	−84.4 (2)	C19—C20—C21—C22	−178.6 (2)
C4—C3—C8—C9	2.7 (3)	C20—C21—C22—C23	0.2 (4)
C2—C3—C8—C9	−105.6 (3)	C21—C22—C23—C24	−0.4 (4)
C4—C3—C8—C7	108.6 (2)	C22—C23—C24—C25	0.2 (4)
C2—C3—C8—C7	0.3 (3)	C23—C24—C25—C20	0.1 (4)
C7—C8—C9—O1	124.9 (3)	C21—C20—C25—C24	−0.3 (3)
C3—C8—C9—O1	−132.5 (3)	C19—C20—C25—C24	178.4 (2)
C7—C8—C9—C10	−44.2 (2)	C6—C2—C1A—C7	−67.4 (3)
C3—C8—C9—C10	58.4 (3)	C3—C2—C1A—C7	46.7 (4)
O1—C9—C10—C11	−128.6 (3)	C11—C7—C1A—C2	66.7 (3)
C8—C9—C10—C11	40.7 (2)	C8—C7—C1A—C2	−46.9 (4)
O1—C9—C10—C5	132.6 (3)	C2—C6—C1B—C11	83.9 (4)
C8—C9—C10—C5	−58.1 (3)	C5—C6—C1B—C11	−40.0 (5)
C4—C5—C10—C9	−2.9 (3)	C7—C11—C1B—C6	−82.9 (4)
C6—C5—C10—C9	104.5 (3)	C10—C11—C1B—C6	40.9 (5)
